# Ways of Coping and Biomarkers of an Increased Atherothrombotic Cardiovascular Disease Risk in Elderly Individuals

**DOI:** 10.1155/2012/875876

**Published:** 2012-07-17

**Authors:** Roland von Känel, Brent T. Mausbach, Joel E. Dimsdale, Paul J. Mills, Thomas L. Patterson, Sonia Ancoli-Israel, Michael G. Ziegler, Susan K. Roepke, Matthew Allison, Igor Grant

**Affiliations:** ^1^Division of Psychosomatic Medicine, Department of General Internal Medicine, Bern University Hospital and University of Bern, 3010 Bern, Switzerland; ^2^Department of Psychiatry, University of California San Diego, La Jolla, CA 92093-0680, USA; ^3^Department of Medicine, University of California San Diego, La Jolla, CA 92103-8341, USA; ^4^San Diego State University Joint Doctoral Program in Clinical Psychology, University of California San Diego, San Diego, CA 92093, USA; ^5^Department of Family and Preventive Medicine, University of California San Diego, La Jolla, CA 92093-0817, USA

## Abstract

*Objective*. To investigate the relationship between coping and atherothrombotic biomarkers of an increased cardiovascular disease (CVD) risk in the elderly. *Methods*. We studied 136 elderly caregiving and noncaregiving men and women who completed the Ways of Coping Checklist to assess problem-focused coping, seeking social support (SSS), blamed self, wishful thinking, and avoidance coping. They had circulating levels of 12 biomarkers measured. We also probed for potential mediator and moderator variables (chronic stress, affect, health behavior, autonomic activity) for the relation between coping and biomarkers. *Results*. After controlling for demographic and CVD risk factors, greater use of SSS was associated with elevated levels of serum amyloid A (*P* = 0.001), C-reactive protein (CRP) (*P* = 0.002), vascular cellular adhesion molecule (VCAM)-1 (*P* = 0.021), and D-dimer (*P* = 0.032). There were several moderator effects. For instance, greater use of SSS was associated with elevated VCAM-1 (*P* < 0.001) and CRP (*P* = 0.001) levels in subjects with low levels of perceived social support and positive affect, respectively. The other coping styles were not significantly associated with any biomarker. *Conclusions*. Greater use of SSS might compromise cardiovascular health through atherothrombotic mechanisms, including elevated inflammation (i.e., serum amyloid A, CRP, VCAM-1) and coagulation (i.e., D-dimer) activity. Moderating variables need to be considered in this relationship.

## 1. Introduction

Coping is undoubtedly one of the most extensively researched concepts in behavioral medicine. How people react to environmental challenges, as well as health-related hardship to reduce psychological distress, is a function of the type of the stressor and of an individual's coping styles which may include thoughts, emotions, and behaviors [1]; the resulting physiological changes may favourably or adversely impact health [2]. However, compared to the literature about the importance of coping for psychological health outcomes, research on coping styles and biological indicators of health is small [3].

Coping styles can be assessed in many ways [4]. Frequently used self-rating tools to measure coping are the Revised Ways of Coping Questionnaire (WOC-R) [5, 6] and the Revised Ways of Coping Checklist (WCCL-R) that derived from the WOC-R [7]. A previous meta-analysis found several subscales from the WOC-R and WCCL-R were associated with psychological and physical health outcomes in nonclinical adult samples, but greater use of seeking social support (SSS) was the only subscale being associated with poor physical health [8]. Individual studies showed that greater use of SSS predicts readmission in patients with coronary heart disease (CHD) [9], microalbuminuria indicative of renal damage in black South African men [10], and reduced stimulated lymphocyte proliferation indicative of cell-mediated immune dysfunction in students [11].

The association between SSS and biological indicators of health may depend upon the quality of the received support [8]. Many of the studies considered in the above mentioned meta-analysis were about relationship-related stressors such as caregiving for a demented spouse. In that case SSS may be distinct from receiving and ultimately perceiving social support because the spouse cannot adequately provide social support. However, it could also be that individuals who ask for support are less healthy, whereby abundant research shows that dementia caregivers have poorer mental and physical health than noncaregiving controls [12]. The SSS scale considers problem-focused (e.g., tangible assistance) and emotion-focused (e.g., sympathy) strategies to interact with others. High level of SSS could actually be maladaptive when a caregiver seeks social support to vent his or her emotional distress. A lack in perceived social support despite high use of SSS might hinder stressed individuals to benefit from the stress buffering effects of social support [13].

The overarching aim of this study was to further elucidate the relationship between coping and biological indicators of health. For this purpose we investigated the relationship between coping strategies, assessed by the WCCL-R and circulating biomarkers of cardiovascular health in a community sample of elderly dementia caregivers and noncaregiving controls. An investigation of these associations in the elderly is clinically important since cardiovascular disease (CVD) becomes more prevalent with aging, and elderly dementia caregivers have a particularly increased risk of developing CVD, particularly CHD [14–16]. We specifically hypothesized that a greater use of SSS would be associated with higher levels of circulating biomarkers that have been linked to CVD, including elevated levels of proinflammatory cytokines, acute phase reactants, cellular adhesion molecules, markers of endothelial dysfunction, and a prothrombotic state [17, 18].

Increases in such biomarkers could be a function of the type of the stressor (e.g., caregiving, low socioeconomic status, life events, health-related problems) with which one needs to cope and of the unique emotional (e.g., negative affect), behavioral (e.g., exercise frequency), and physical (e.g., sympathetic activation) responses to such a stressor. For instance, if caregivers seek out for social support strongly, but will not receive it, this might elicit depressed mood that has been associated with a proinflammatory state [19]. Thus, in several exploratory analyses, we also tested whether (a) chronic stress, (b) affect, (c) health behavior, and (d) autonomic activity would mediate or moderate the relationship between coping styles and levels of circulating biomarkers.

## 2. Methods

### 2.1. Study Participants

The University of California San Diego (UCSD) Institutional Review Board approved the study protocol and all participants provided written consent. For the present study, we analyzed cross-sectional data obtained at study entry of the UCSD “Alzheimer's Caregiver Study” investigating effects of dementia caregiving stress on health of elderly spousal caregivers. Participants were recruited through referrals from the UCSD Alzheimer's Disease Research Center, community support groups and agencies serving caregivers, local senior citizen health fairs, and referrals from other participants. Inclusion criteria were being ≥55 years old, married, and dwelling in the community with a spouse. Exclusion criteria were presence of any major illnesses (e.g., cancer), severe hypertension (BP > 200/120 mmHg), or treatment with medication that were known to affect biomarkers of interest (i.e., oral anticoagulants, nonselective beta blockers, steroids). Caregivers had to provide primary care for a spouse with a physician-based diagnosis of Alzheimer's disease. Noncaregivers were recruited in the same communities to yield a gender- and age-equated comparison group. By definition, spouses of these noncaregivers did not require care for a serious medical condition. Other exclusion criteria were as for caregivers above. 

Out of 186 enrolled subjects (126 caregivers, 60 noncaregivers), 151 had complete data for the 12 assessed biomarkers. One subject each missed data on coping, body mass index (BMI), and dyslipidemia, and 12 subjects missed data on norepinephrine (NEPI) levels. This yielded a sample of 136 subjects (93 caregivers, 43 noncaregivers) with a complete dataset for the present investigation allowing us to compute full linear regression approach. The 45 subjects with incomplete data did not significantly differ from the 136 subjects with complete data for sociodemographic factors, CVD risk factors, caregiving status, and ways of coping. All participants were interviewed in their homes using questionnaires to assess demographic factors, mood states, stressors, and health status. 

### 2.2. Sociodemographic, Health, and Psychometric Measures

#### 2.2.1. Demographic Factors

We collected information on gender, age, and years of education to define socioeconomic status. 

#### 2.2.2. Body Mass Index

 We asked participants for their weight and height to calculate BMI.

#### 2.2.3. Dyslipidemia

Plasma low-density lipoprotein cholesterol (LDL-C) and high-density lipoprotein cholesterol (HDL-C) were determined by standard methodology at the clinical chemistry laboratories at the UCSD medical center. We computed the LDL-C/HDL-C ratio with a higher ratio indicating greater dyslipidemia.

#### 2.2.4. Blood Pressure and Heart Rate

 Using a noninvasive Microlife blood pressure (BP) monitor, three BP and heart rate measurements were collected by the research nurse over a 15-minute resting period. The participant's mean systolic resting BP was used for the analysis because it confers higher CVD risk than diastolic BP in individuals over 50 years of age [20]. 

#### 2.2.5. Smoking Status

Smoking status was defined in terms of ever (i.e., former plus current) smoker versus never smoker (only one participant was a current smoker).

#### 2.2.6. Health Problems

 Participants were provided a list with 17 health problems they might currently have or that a doctor had informed them of having. Positive items were added to one *number of health problems*. We also formed separate categories *for diabetes and any cardiovascular disease* that included myocardial infarction, congestive heart failure, angina, additional heart diseases, and stroke (but not systemic hypertension).

#### 2.2.7. Alcohol Consumption

The amount of alcohol consumption was assessed using a score that considered the number of days subjects had at least one alcoholic drink and the number of alcoholic drinks they usually drank on these days considering the last month.

#### 2.2.8. Physical Activity

The Rapid Assessment of Physical Activity (RAPA) scale was used to assess the amount of light, moderate, and strenuous physical activities, including strength and flexibility exercises, in a typical week (total score 0–6) [21].

#### 2.2.9. Sleep Quality

The Pittsburgh Sleep Quality Index was used to assess subjective sleep quality, sleep duration, sleep latency, sleep disturbances, sleep efficacy, use of sleep medication, daytime dysfunction (global score between 0 and 21; higher scores indicate poorer sleep quality) [22].

#### 2.2.10. Coping

Participants completed the WCCL-R [7] to assess problem-focused coping (PFC; 15 items), seeking social support (SSS; 6 items), blaming one's self (BS; 3 items), wishful thinking (WT, 8 items), and avoidance coping (AC, 10 items). Participants were asked to rate on a four point scale the degree to which they used 42 coping strategies in dealing with stressful situations that may arise in marriages (0 = never used, 1 = used somewhat, 2 = used quite a bit, 3 = used a great deal). Typical strategies are “Got professional help and did what they recommended” for PFC, “Talked to someone to find out about the situation” for SSS, “Blamed yourself” for BS, “Hoped a miracle would happen” for WT, and “Avoided being with people in general” for AC. 

#### 2.2.11. Affect

We used the Positive and Negative Affect Scale comprising 10 items per mood scale covering the past few weeks on a 5-point scale (1 = very slightly or not at all, 5 = extremely; total score between 10 and 50) [23].

#### 2.2.12. Life Events

We used the Life Events Survey which assesses how many of a list of 34 events had occurred to the participant or a close relative or friend in the past year [24].

#### 2.2.13. Social Support

The 8-item Social Support Scale was used to assess help and support participants received from friends and relatives [25]. Responses were rated on a 4-point scale: 1 = strongly disagree, 4 = strongly agree; overall scores were between 8 and 32.

#### 2.2.14. Norepinephrine Levels

A highly sensitive catechol-o-methyltransferase (COMT)-based radioenzymatic assay was performed to determine plasma NEPI [26].

### 2.3. Biomarkers

Blood was collected in the participant's home at 10:30 AM. In order not to interfere with study participants daily routine, fasting state was not a prerequisite but was treated as a control variable. Plasma was stored at −80°C until analyzed. Concentrations of biomarkers were determined in duplicates using commercially available enzyme-linked immunosorbent assays per the manufacturers' instructions (Meso Scale Discovery, Gaithersburg, MD: C-reactive protein (CRP), tumor necrosis factor (TNF)-*α*, interleukin (IL)-6, IL-8, interferon (IFN)-*γ*, serum amyloid A (SAA), soluble intercellular adhesion molecule (sICAM)-1, soluble vascular cellular adhesion molecule (sVCAM)-1; Quantikine, R&D Systems, Minneapolis, MN: endothelin (ET)-1; Asserachrom Stago, Asnières, France: von Willebrand factor (VWF), plasminogen activator inhibitor (PAI)-1, D-dimer).

We selected these 12 biomarkers because all of them are variously involved in the initiation, progression, and clinical manifestation of atherothrombotic CVD. They specifically cover atherosclerotic processes related to inflammation (proinflammatory cytokines: TNF-*α*, IL-6, IL-8, IFN-*γ*; acute phase reactants: SAA, CRP), cellular adhesion (sICAM-1, sVCAM-1), endothelial dysfunction (ET-1, VWF), and thrombosis (PAI-1, D-dimer). A large body of literature suggests that elevated circulating levels of these biomarkers predict the risk of incident CHD as well as poor prognosis in patients with established CHD [27–36]. 

### 2.4. Data Analysis

Data were analyzed using PASW 18.0 statistical software package (SPSS Inc., Chicago, IL, USA) with *P* ≤ 0.05 (two-tailed). Normality of the distribution of dependent variables (biomarkers) was verified using the Kolmogorov-Smirnov test. Accordingly, IL-8, IFN-*γ*, SAA, CRP, sICAM-1, ET-1, VWF, PAI-1, and D-dimer values were all logarithmically transformed, and Blom normal scores were computed for IL-6 and sVCAM-1 values. Pearson correlation coefficients were calculated to estimate the relation between two variables. Group comparisons used independent *t*-test for continuous variables. Pearson *χ* ^2^ test and Fisher's exact test, where appropriate, were conducted for categorical measures.

To test for a relation between coping and biomarkers, we first employed multivariate analysis of covariance (MANCOVA) to test whether each of the five coping scales would be significantly associated with the group of the 12 biomarkers as a whole applying Bonferroni correction (*P* < 0.010 for 5 coping strategies). Control variables were selected a priori as they may potentially affect biomarker levels: age, gender, BMI, LDL-C/HDL-C ratio, systolic BP, smoking status, diabetes, any CVD, and fasting state. We further tested for potential mediator and moderator variables (i.e., chronic stress, affect, health behavior, autonomic activity) of the relation between coping and individual biomarkers using linear regression analysis. Linear independent variables were centered at their means and binary variables were centered as +0.5 and −0.5 [37]. Effect sizes from the regression models are expressed as partial correlation coefficients (*r*). In case of a significant interaction, we applied the Holmbeck method to test whether high versus low levels in the moderator variable, defined as +1 SD and −1 SD, respectively, would alter the strength of the association between coping and the biomarker [38]. A potential mediator was defined as an independent variable that if added to a model would render a significant relation between coping scales and biomarkers nonsignificant. Cook's distance was used to verify the absence of outliers. 

## 3. Results

### 3.1. Characteristics of Study Participants

Table 1 shows the characteristics of study participants. Compared to noncaregiving control subjects, caregivers showed more CVD and health problems, poorer sleep quality, and lower physical activity. Caregivers also showed more negative affect, SSS, and WT, but less perceived social support than noncaregivers. There were no significant group differences in the concentrations of any of the biomarkers. SSS and perceived social support showed a direct but weak association (*r* = 0.18, *P* = 0.036). 

### 3.2. Relationship between Coping Scales and Biomarkers as a Group

We first performed MANCOVA to test for a significant relationship between each of the five coping scales and the group of 12 biomarkers as a whole. All analyses controlled for age, gender, BMI, LDL-C/HDL-C ratio, systolic BP, smoking status, diabetes, any CVD, and fasting state. We found a significant relationship between SSS and the group of biomarkers as a whole (*F*_12,114_ = 2.46, *P* = 0.007; partial *η* ^2^ = 0.206). In contrast, there were no significant relationships between the group of biomarkers and PFC (*P* = 0.058), BS (*P* = 0.86), WT (*P* = 0.36), and AC (*P* = 0.47). In the model for SSS, age (*P* < 0.001; partial *η* ^2^ = 0.258), BMI (*P* < 0.001; partial *η* ^2^ = 0.262), and fasting state (*P* = 0.043; partial *η* ^2^ = 0.166) were also significantly related to the entire group of biomarkers.

### 3.3. Relationship between Seeking Social Support and Individual Biomarkers

In MANCOVA, SSS showed significant relationships with IL-8 (*P* = 0.003), SAA (*P* = 0.001), CRP (*P* = 0.012), sVCAM-1 (*P* = 0.021), and D-dimer (*P* = 0.032). The relationships with TNF-*α* (*P* = 0.45), IL-6 (*P* = 0.65), INF-*γ* (*P* = 0.79), sICAM-1 (*P* = 0.065), ET-1 (*P* = 0.70), VWF (*P* = 0.30), and PAI-1 (*P* = 0.22) were not significant.

The multivariate linear regression models for individual biomarkers are shown in Table 2. Greater use of SSS was significantly correlated with decreased levels of IL-8 (*r* = −0.26) on the one hand and elevated levels of SAA (*r* = 0.29), CRP (*r* = 0.22), sVCAM-1 (*r* = 0.20), and D-dimer (*r* = 0.19) on the other. Of the covariates, greater age was significantly associated with higher D-dimer, greater BMI was associated with higher SAA and CRP, the latter also being directly related to systolic BP. Participants who were assessed in the fasting state had lower IL-8 than those assessed nonfasting.

### 3.4. Mediators of the Relation between Seeking Social Support and Biomarkers

All meditational analyses were adjusted for age, gender, BMI, LDL-C/HDL-C ratio, systolic BP, smoking status, diabetes, any CVD, and fasting state. Chronic stress (caregiving, socioeconomic status, life events, health problems), affect (negative affect, positive affect, perceived social support), health behaviors (alcohol consumption, physical activity, sleep), and autonomic activity (NEPI, heart rate) did not emerge as significant mediators of the association of SSS with IL-8, SAA, CRP, sVCAM-1, and D-dimer (data not shown). 

### 3.5. Moderators of the Relation between Seeking Social Support and Biomarkers

All interaction effects were adjusted for age, gender, BMI, LDL-C/HDL-C ratio, systolic BP, smoking status, diabetes, any CVD, fasting state, SSS (main effect), and the respective moderator variable (main effect).

**Chronic Stress**

There were significant interactions between SSS and caregiver status for D-dimer (*r* = −0.19, *P* = 0.037) and between the number of life events and IL-8 (*r* = 0.22, *P* = 0.015). Greater use of SSS was associated with elevated D-dimer levels in noncaregivers (*r* = 0.36, *P* = 0.036), while SSS and D-dimer showed no association in caregivers (*r* = −0.04, *P* = 0.75). Greater use of SSS was associated with decreased IL-8 levels in subjects with a small number of life events (*r* = −0.33, *P* < 0.001), whereas SSS and IL-8 showed no association in those with a high number of life events (*r* = −0.07, *P* = 0.046). Socioeconomic status (years of education) (*P* values > 0.26) and health-related stress (the number of health problems) (*P* values > 0.20) did not both emerge as moderator variables for any biomarker.

**Affect**

There were significant interactions between SSS and positive affect for CRP (*r* = −0.11, *P* = 0.020) and between SSS and perceived social support for sVCAM-1 (*r* = −0.25, *P* = 0.006). Greater use of SSS was associated with elevated levels of sVCAM-1 in those with low levels of social support (*r* = 0.32, *P* < 0.001), whereas no association emerged between SSS and sVCAM-1 in those with high levels of social support (*r* = −0.01, *P* = 0.89). In addition, greater use of SSS was associated with elevated CRP levels in those with low levels of positive affect (*r* = 0.30, *P* = 0.001), while SSS showed no association with CRP in those with high levels of positive affect (*r* = 0.03, *P* = 0.75). Negative affect did not interact with SSS in predicting any biomarker (*P*-values > 0.52).

**Health Behavior**

There were no significant interactions between SSS on the one hand and physical activity (*P*-values > 0.45), alcohol intake (*P* values > 0.20), and sleep quality (*P* values > 0.25) on the other for any biomarker.

**Autonomic Nervous System Function**

There was a significant interaction between NEPI and SSS for IL-8 (*r* = −0.40, *P* < 0.001). Greater use of SSS was associated with decreased levels of IL-8 in subjects with high levels of NEPI (*r* = −0.46, *P* < 0.001), whereas no association between SSS and IL-8 was seen in those with low levels of NEPI (*r* = 0.10, *P* = 0.27). Heart rate did not significantly interact with SSS in predicting any biomarker (*P* values > 0.17).

## 4. Discussion

We found that greater use of SSS was associated with elevated levels of several circulating biomarkers, all of which are proxy measures of an increased risk of atherothrombotic CVD in elderly community-dwelling subjects. This relationship was independent of sociodemographic and CVD risk factors that are known to affect biomarker levels. Specifically, we found greater use of SSS to be associated with increased levels of SAA, CRP, sVCAM-1, and D-dimer. We did not find that PFC, BS, WT, and AC were significantly related to biomarkers. The finding that greater use of SSS is associated with elevated levels of biomarkers of atherothrombotic risk is a novel one. Moreover, this observation is consistent with a previous meta-analysis showing that greater use of SSS was associated with negative physical health outcomes [8], including a higher risk of hospital readmission in patients with atherothrombotic CVD [9]. Greater use of SSS has also been associated with a greater prevalence of microalbuminuria [10], another risk factor for atherothrombotic CVD. Although our cross-sectional data do not allow causal inferences among the observed relationships, the pattern of results is consistent with the notion that greater use of SSS may increase atherothrombotic CVD risk. The finding that greater use of SSS related to elevations in multiple biomarkers strengthens the validity of the biological pathway that may be implicated in SSS's links to poor cardiovascular health.

A variety of mechanisms might be involved in this pathway. The acute phase reactant SAA is expressed by human adipocytes and atherosclerotic lesions and may play a critical role in local and systemic inflammation by linking obesity and atherosclerosis [39]. CRP is also expressed in the liver and in smooth muscle cells within diseased atherosclerotic arteries. CRP has been implicated in multiple aspects of atherogenesis. It particularly amplifies inflammatory responses and induces expression of cellular adhesion molecules (e.g., ICAM-1, VCAM-1), which mediate adhesion of leukocytes to the vascular endothelium, as well as a decrease in nitric oxide production, thereby promoting vascular constriction [40]. Circulating concentrations of soluble CAMs such as sVCAM-1 are markers of the inflammatory cascade and of endothelial cell injury and dysfunction. Soluble CAMs are actively involved in many of the stages of atheroma development from initial leukocyte recruitment to eventual rupture of the unstable atherosclerotic plaque [41]. In addition, the prothrombotic measure D-dimer indicates coagulation activation and in vivo fibrin formation and lysis in circulating blood, and its localization within the human intima suggests it may have atherogenic properties [42]. 

To gain more insight into the mechanisms linking greater use of SSS with biomarkers, we performed a series of exploratory analyses in which we tested whether chronic stress, affect, health behaviour, and autonomic activity might possibly act as mediator or moderator variables. None of these variables were suggested as mediators; however, there may be other mediators that are relevant, but not tested in our study. For instance, a life-time approach to experienced psychosocial stress (i.e., considering stress burden for longer than one year), inclusion of daily hassles, account of dietary habits, and more thorough autonomic measures (e.g., heart rate variability) might have also be important to investigate.

We found that the seemingly counterintuitive inverse association between greater use of SSS and decreased levels of the proinflammatory marker IL-8 was moderated by life events and NEPI. Specifically, greater use of SSS was associated with decreased IL-8 in subjects with few life events, although it would seem that cardiovascular health might particularly draw benefit from such an effect if life stress is high. Moreover, greater use of SSS was also associated with decreased IL-8 when NEPI levels were high. As NEPI increases the IL-8 release from the endothelium [43], we speculate that SSS interferes with NEPI-related IL-8 release through a mechanism that remains to be elucidated. Of equal interest were the moderating roles of positive affect and perceived social support for CRP and sVCAM-1, respectively, suggesting that greater use of SSS is particularly related to increased atherothrombotic risk if factors of positive mental health are low. Specifically, greater use of SSS was associated with elevated CRP levels in subjects with low level of positive affect (i.e., in those feeling desperate); this is in accordance with the notion that positive affect is associated with psychobiological processes that may be partly responsible for the protective effects of positive affect on physical health outcomes [44], including lower risk of incident CHD [45]. Moreover, greater use of SSS was associated with elevated sVCAM-1 in subjects with low level of social support (i.e., in those feeling frustrated because not getting support in spite of seeking it); this concurs with the concept of perceived social support acting as a stress buffer [13], thereby reducing the risk of incident CHD [46].

These interaction effects suggest that help seeking is not uniformly bad for cardiovascular health when assessed through biomarkers, so elderly individuals clearly should not be discouraged from seeking help. In fact, entire interventions are based on the idea that for instance dementia caregivers need to seek out additional support from their social environment in order to better cope with caregiving burden [47]. This might partially explain why there was no association between SSS and D-dimer levels in caregivers, whereas greater use of SSS was associated with elevated D-dimer levels in noncaregivers. However, even though caregivers reported greater use of SSS than noncaregivers, we found little evidence that caregiver status substantially moderated the association of greater use of SSS with elevated SAA, CRP, and sVCAM-1 on the one hand and with decreased IL-8 on the other. It is possible that copings styles other than SSS are more important in not only maintaining but also impacting on physical health in dementia caregivers. For instance, we previously found that caregivers high in personal mastery (i.e., a person's belief that he/she can control the circumstances of his/her life) had less NEPI reactivity to acute mental stress [48] but greater *β*2-adrenergic receptor sensitivity indicative of better immune function over a followup of 5 years [49] compared to caregivers low in personal mastery.

From a clinical perspective, our data may imply that encouragement of elderly individuals by clinicians about seeking out support to possibly improve their cardiovascular health needs to consider that not all friends and family will be perceived as sources of support in which case greater use of SSS might not be helpful if not harmful. This might particularly be the case for those elderly low in positive affect and low in perceived social support. One recommendation could be that elderly individuals are advised to disengage in time from seeking support from certain members of their social network should they realize they will not receive the expected amount and quality of emotional and instrumental help. Whether such a strategy would be effective in improving cardiovascular health might be validated in an intervention study aimed at changing coping behavior where a decrease in the use of SSS should be associated with a concomitant decrease in SAA, CRP, and D-dimer levels. However, these implications must consider that the interactions between SSS and some covariates were not observed for all biomarkers. Moreover, the number of exploratory analyses conducted was substantial. Therefore, the clinical conclusions and recommendations form these secondary findings should be made with caution. 

We discuss three limitations of our study. Firstly, cross-sectional investigations capture only a snapshot of biobehavioral processes. Coping processes are particularly actuated when challenge occurs at which time biomarker response would seem to be greatest. Therefore, experimental induction of acute stressful situations might be a more promising way to investigate which coping styles are either functional or dysfunctional in terms of moderating the stress response of biomarkers. Secondly, biomarkers are intermediate endpoints of CVD. Although similar to that explained by age and BMI, the variances in biomarkers that were explained by SSS ranged 4–9%. Whether this effect size translates into a clinically relevant increase in atherothrombotic CVD risk needs to be seen in a longitudinal study. Thirdly, our results stem from elderly with on average good physical and mental health, two thirds of whom were dementia caregivers. Hence, the findings may not be generalizable to younger populations or elderly with greater prevalence of CVD and frailty.

## 5. Conclusions

The findings from our study suggest that elderly community-dwelling individuals with increased use of SSS may have elevated circulating levels of a range of biomarkers that have been associated with an increased risk of atherothrombotic CVD. Several moderating variables relating to the type of chronic stress, affect, and sympathomedullary activity need to be considered in this relationship. If replicated, these results might offer promising avenues for interventions focused on healthy coping strategies that might also improve cardiovascular health in the elderly. These might include provision of better practical and emotional support, as well as cognitive-behavioral interventions to modify perceptions of burden and support.

## Figures and Tables

**Table 1:** Participant characteristics.

Variables	All participants (*n* = 136)	Caregivers (*n* = 93)	Noncaregivers (*n* = 43)	*P*
Age (years)	74.5 ± 7.7	74.6 ± 8.4	74.5 ± 6.2	0.937
Women (%)	67.6	67.7	67.4	0.972
Education (years)	15.4 ± 3.2	15.3 ± 3.1	15.5 ± 3.4	0.722
Body mass index (kg/m^2^)	26.4 ± 5.2	26.7 ± 4.9	25.9 ± 5.9	0.447
LDL-C/HDL-C ratio	2.2 ± 0.9	2.2 ± 0.8	2.2 ± 1.0	0.904
Systolic blood pressure (mm Hg)	134 ± 15	135 ± 15	132 ± 15	0.432
Heart rate (beats/min)	66 ± 10	66 ± 10	66 ± 9	0.802
Ever smoker (%)	40.4	43.0	34.9	0.369
Diabetes (%)	9.6	12.9	2.3	0.062
Any cardiovascular disease (%)	14.7	19.4	4.7	0.035
Number of health problems	3.1 ± 1.8	3.5 ± 1.9	2.5 ± 1.4	0.001
Alcohol consumption (score)	5.4 ± 5.8	5.4 ± 5.7	5.5 ± 6.1	0.908
Rapid Assessment of Physical Activity (score)	3.6 ± 1.6	3.3 ± 1.6	4.1 ± 1.4	0.005
Pittsburgh Sleep Quality Index	6.1 ± 3.4	6.7 ± 3.5	4.8 ± 2.5	<0.001
Problem-focused coping (score)	22.2 ± 7.2	22.4 ± 6.8	21.9 ± 8.1	0.702
Seeks social support (score)	6.3 ± 4.2	6.9 ± 3.9	5.2 ± 4.5	0.027
Blamed self (score)	2.3 ± 1.9	2.3 ± 2.0	2.1 ± 1.8	0.626
Wishful thinking (score)	7.2 ± 4.8	7.8 ± 4.9	5.8 ± 4.3	0.020
Avoidance coping (score)	7.2 ± 4.0	7.5 ± 4.1	6.3 ± 3.6	0.098
Negative affect (score)	16.7 ± 6.3	17.9 ± 6.2	14.0 ± 5.7	0.001
Positive affect (score)	33.6 ± 7.5	31.8 ± 7.4	37.4 ± 6.4	<0.001
Number of life events	4.7 ± 3.3	5.0 ± 3.3	4.0 ± 3.1	0.108
Perceived social support (score)	26.6 ± 3.9	25.9 ± 3.9	28.0 ± 3.5	0.004
Fasting state (%)	15.4	12.9	20.9	0.307
Norepinephrine (pg/mL)	471 ± 210	472 ± 218	467 ± 193	0.885
Tumor necrosis factor-*α* (pg/mL)	5.76 (4.11–7.64)	5.79 (4.12–7.70)	5.41 (4.09–7.66)	0.603
Interleukin-6 (pg/mL)	1.12 (0.80–1.67)	1.07 (0.84–1.53)	1.30 (0.75–1.86)	0.189
Interleukin-8 (pg/mL)	6.91 (4.22–9.54)	6.88 (4.15–9.11)	7.07 (4.25–10.1)	0.267
Interferon-*γ* (pg/mL)	1.61 (0.96–2.39)	1.59 (1.06–2.41)	1.74 (0.80–2.29)	0.348
Serum amyloid A (mg/mL)	2.44 (1.13–7.39)	2.53 (1.14–7.30)	2.09 (1.01–8.06)	0.933
C-reactive protein (mg/mL)	1.42 (0.81–3.92)	1.49 (0.85–4.16)	1.24 (0.69–3.05)	0.422
Intercellular adhesion molecule-1 (ng/mL)	295 (217–489)	275 (208–486)	316 (224–490)	0.480
Vascular cellular adhesion molecule-1 (ng/mL)	541 (369–950)	545 (365–956)	539 (397–930)	0.842
Endothelin-1 (pg/mL)	1.10 (0.81–1.40)	1.08 (0.80–1.40)	1.15 (0.84–1.42)	0.514
von Willebrand Factor (%)	158 (83–270)	156 (92–269)	144 (75–287)	0.457
D-dimer (ng/mL)	630 (452–954)	671 (487–980)	613 (415–957)	0.300
Plasminogen activator inhibitor-1 (ng/mL)	25.9 (15.6–43.3)	29.1 (16.7–47.8)	21.1 (12.6–33.6)	0.149

Nonnormal distribution (even after log transformation)/all biomarkers (IVs) were log transformed/shown as median (IQR) Fischer Definition?

**Table 2:** Multivariate linear regression model for the relationship between seeking social support and individual biomarkers.

Entered variables	Interleukin-8		Serum amyloid A		C-reactive protein		Soluble VCAM-1		D-dimer	
	Partial corr.	*P* value	Partial corr.	*P* value	Partial corr.	*P* value	Partial corr.	*P* value	Partial corr.	*P* value
Age	0.036	0.688	−0.001	0.989	0.041	0.649	0.124	0.164	0.368	**<0.001**
Female gender	0.078	0.385	0.142	0.111	0.167	0.061	−0.064	0.471	0.169	0.058
Body mass index	0.053	0.552	0.203	**0.022**	0.215	**0.015**	0.123	0.169	0.014	0.873
LDL-C/HDL-C ratio	−0.017	0.846	−0.008	0.925	0.111	0.214	0.026	0.774	−0.006	0.951
Systolic blood pressure	−0.065	0.469	0.158	0.077	0.175	**0.049**	0.067	0.4522	0.046	0.604
Ever smoker	0.016	0.860	0.009	0.924	0.062	0.492	−0.013	0.884	0.059	0.508
Diabetes	0.076	0.398	−0.078	0.381	−0.095	0.288	−0.113	0.207	0.015	0.867
Any cardiovascular disease	−0.105	0.240	−0.076	0.395	−0.080	0.369	0.044	0.623	0.094	0.295
Fasting state	−0.206	**0.020**	0.033	0.716	0.013	0.881	0.097	0.280	−0.051	0.566
Seeking social support	−0.264	**0.003**	0.289	**0.001**	0.223	**0.012**	0.204	**0.021**	0.190	**0.032**

Model statistic	*R* ^2^ = 0.139		*R* ^2^ = 0.186		*R* ^2^ = 0.189		*R* ^2^ = 0.098		*R* ^2^ = 0.227	
	*F*_10,125_ = 2.02,		*F*_10,125_ = 2.86,		*F*_10,125_ = 2.92,		*F*_10,125_ = 1.36,		*F*_10,125_ = 3.68,	
	*P* = 0.036		*P* = 0.003		*P* = 0.003		*P* = 0.209		*P* < 0.001	

Partial corr.: partial correlation coefficient, significant *P* values are in bold.
